# Evaluation of low-density SNP panels and imputation for cost-effective genomic selection in four aquaculture species

**DOI:** 10.3389/fgene.2023.1194266

**Published:** 2023-05-11

**Authors:** Christina Kriaridou, Smaragda Tsairidou, Clémence Fraslin, Gregor Gorjanc, Mark E. Looseley, Ian A. Johnston, Ross D. Houston, Diego Robledo

**Affiliations:** ^1^ The Roslin Institute and Royal (Dick) School of Veterinary Studies, University of Edinburgh, Edinburgh, United Kingdom; ^2^ Global Academy of Agriculture and Food Systems, University of Edinburgh, Edinburgh, United Kingdom; ^3^ Xelect Ltd., St Andrews, Scotland, United Kingdom; ^4^ Benchmark Genetics, Penicuik, United Kingdom

**Keywords:** selective breeding, imputation, genomic prediction, aquaculture, fish, bivalve

## Abstract

Genomic selection can accelerate genetic progress in aquaculture breeding programmes, particularly for traits measured on siblings of selection candidates. However, it is not widely implemented in most aquaculture species, and remains expensive due to high genotyping costs. Genotype imputation is a promising strategy that can reduce genotyping costs and facilitate the broader uptake of genomic selection in aquaculture breeding programmes. Genotype imputation can predict ungenotyped SNPs in populations genotyped at a low-density (LD), using a reference population genotyped at a high-density (HD). In this study, we used datasets of four aquaculture species (Atlantic salmon, turbot, common carp and Pacific oyster), phenotyped for different traits, to investigate the efficacy of genotype imputation for cost-effective genomic selection. The four datasets had been genotyped at HD, and eight LD panels (300–6,000 SNPs) were generated *in silico*. SNPs were selected to be: i) evenly distributed according to physical position ii) selected to minimise the linkage disequilibrium between adjacent SNPs or iii) randomly selected. Imputation was performed with three different software packages (AlphaImpute2, FImpute v.3 and findhap v.4). The results revealed that FImpute v.3 was faster and achieved higher imputation accuracies. Imputation accuracy increased with increasing panel density for both SNP selection methods, reaching correlations greater than 0.95 in the three fish species and 0.80 in Pacific oyster. In terms of genomic prediction accuracy, the LD and the imputed panels performed similarly, reaching values very close to the HD panels, except in the pacific oyster dataset, where the LD panel performed better than the imputed panel. In the fish species, when LD panels were used for genomic prediction without imputation, selection of markers based on either physical or genetic distance (instead of randomly) resulted in a high prediction accuracy, whereas imputation achieved near maximal prediction accuracy independently of the LD panel, showing higher reliability. Our results suggests that, in fish species, well-selected LD panels may achieve near maximal genomic selection prediction accuracy, and that the addition of imputation will result in maximal accuracy independently of the LD panel. These strategies represent effective and affordable methods to incorporate genomic selection into most aquaculture settings.

## 1 Introduction

Aquaculture has been the fastest-growing food production sector in recent decades, with a 609% rise in the total annual output from 1990 to 2020 (FAO, 2022). This growth has revolutionised the supply of seafood products across the planet, providing nutritious seafood to a growing human population and significantly contributing to meeting food security objectives in many regions. However, the development of aquaculture in different countries has been uneven, and seafood production still needs to be increased to ensure food security and reduce the effect of fishing on wild populations, offsetting the environmental impacts of overexploitation (Cottrell et al., 2021).

In 2016 over 95% of the global aquaculture output originated from low and middle-income countries (Stentiford et al., 2020). The rapid expansion of aquaculture in these countries is primarily due to the adoption of aquaculture by small and medium-sized enterprises, but there are still challenges that hold back the development of smaller aquaculture settings (Kumar et al., 2018; FAO, 2020). A significant restriction is the lack of well-managed breeding programmes for directional selection and improvement of desirable traits. In addition, the establishment of breeding programmes for small farms is expensive. Therefore, where basic breeding programmes exist, they lag behind in the implementation of the available genomic tools utilised by modern breeding programmes due to the high cost compared to their relatively small production. The use of genomics can improve selection intensity and breeding value prediction accuracy, particularly for traits not possible to measure directly on selection candidates. In turn, this can then lead to a more efficient production, benefiting the entire supply chain, which is essential to unlock the potential of aquaculture stocks and ensure food security (Houston et al., 2020; FAO, 2022).

Genomic selection uses genetic markers to more accurately predict the breeding values of individuals compared to pedigree-based approaches, leading to higher rates of genetic gain and better management of inbreeding (Houston et al., 2020; Boudry et al., 2021; Regan et al., 2021). Despite its potential, genomic selection has only been implemented in the most advanced aquaculture sectors, and only for a small number of aquatic species, such as Atlantic salmon, rainbow trout, American catfish, whiteleg shrimp or Nile tilapia (Lillehammer et al., 2020; Yáñez et al., 2020; Boudry et al., 2021; Houston et al., 2022). One of the barriers to the widespread adoption of genomic selection is the high cost of genotyping. Genotyping can be prohibitively expensive for small and medium aquaculture operations, making it more challenging for them to adopt genomic selection practises (Boudry et al., 2021). For these industries to benefit from genomic selection, low-cost genotyping strategies that do not significantly compromise the prediction accuracy of breeding values are required.

Several studies have looked into the use of low-density (LD) SNP panels as a cost-effective alternative, with only a few thousands or even hundreds of SNPs used for genomic selection, in contrast to high-density (HD) panels, usually containing tens of thousands SNPs. Generally, studies on aquaculture species have reported that SNP densities can be reduced from tens of thousands to thousands without a significant loss of prediction accuracy (Tsai et al., 2016; Palaiokostas et al., 2018; 2019; Robledo et al., 2018; Yoshida et al., 2019; Gutierrez et al., 2020; Kriaridou et al., 2020; Tsairidou et al., 2020; Al-Tobasei et al., 2021). Additionally, complementary strategies such as genotype imputation can be used to further reduce the cost and improve the accuracy of low-cost genomic selection.

Genotype imputation is a method that can be used to predict missing genotypes in an individual based on the genotypes of other individuals of the same species. A common imputation strategy is to use a group of individuals genotyped with a HD panel (reference population) to infer the missing genotypes of other individuals (target population) genotyped with a LD panel, which is composed of a subset of markers from the HD panel (Marchini and Howie, 2010; Sargolzaei et al., 2010). The reference and target populations need to be related to some degree as imputation relies on linkage and linkage disequilibrium within those populations. The general idea of genotype imputation is that related individuals share long haplotype blocks (set of markers in linkage disequilibrium segregating together). These haplotype blocks are broken by recombination events occurring from one generation to the next; hence two animals will share longer haplotypes the more related they are.

Imputation algorithms can use a combination of population and pedigree-based methods (Browning, 2008; Bouwman et al., 2014; Sargolzaei et al., 2014; Wang et al., 2016; Antolín et al., 2017; Lashmar et al., 2019; Phocas, 2022). FImpute (Sargolzaei et al., 2014) and AlphaImpute (Whalen and Hickey, 2020) are popular algorithms developed for animals and plants, combining population and pedigree-based imputation methods. Population-based methods utilise linkage disequilibrium information between markers in various ways. Generally, they use Hidden Markov Model (HMM) approaches to model genotype and underlying haplotype variation relying on population-wide linkage disequilibrium between markers (short shared haplotypes) (Sargolzaei et al., 2014; Whalen et al., 2018). Pedigree-based methods incorporate information from linkage and pedigree relationships for imputation. These methods take advantage of the long-haplotypes shared by closely related individuals, such as parent-offspring or full-sibs, as well as using Mendelian inheritance rules to infer missing genotypes (Antolín et al., 2017). Pedigree information increases in importance as the LD panel becomes sparser, because it enables capturing the long-range haplotype blocks shared between relatives. Studies where imputation is applied to a population of related individuals (family studies) are more powerful and effective in identifying low-frequency variants (Sargolzaei et al., 2014; Liu et al., 2019). The choice of software can also impact the results; different algorithms make use of the available information differently, so the optimal imputation software may differ depending on the population of interest.

In addition to the imputation method, there are several other factors affecting genotype imputation accuracy, namely, SNP minor allele frequency (MAF), the selection of SNPs for the LD panel (number of SNPs and their chromosomal distribution), the number of individuals in the reference population and the population structure. MAF significantly impacts imputation accuracy for all imputation methods; as MAF increases, the accuracy of imputation of the minor allele increases (Wang et al., 2016). Imputation of rare alleles is important because variants with low frequency may have large effects, linked to the “missing heritability” in some complex traits (Manolio et al., 2009; Sargolzaei et al., 2014; Gonzalez-Recio et al., 2015). The size of the reference population also affects imputation; the greater the number of individuals in the reference panel, and the more closely related they are to the target individuals, the more accurate is genotype imputation (Garcia et al., 2022). Finally, one aspect that requires further investigation is the impact of SNP selection strategy for the LD panel. Various methods have been proposed for the design of LD SNP panels, such as: i) randomly selected SNPs across the genome or within the chromosome (Tsairidou et al., 2020), ii) evenly spaced according to position and chromosome size (Yoshida et al., 2021), iii) based on linkage disequilibrium patterns (Yoshida et al., 2021), iv) selection of highly polymorphic SNPs explaining most of the phenotypic variance of a trait (Aliloo et al., 2018; Wu et al., 2020), v) or even the design of multi-trait-specific SNP panels (He et al., 2018) and family-specific SNP panels (Whalen et al., 2019). These studies have shown that for some traits, the SNP selection method for the LD panel plays an important role.

Several studies have compared the performance of imputation software and the different parameters affecting genotype imputation in human, plant and livestock populations. However, aquaculture broodstock populations are typically comprised of relatively few (but large) full and half sib families, with limited population structure and, as such, might be expected to show a different response to imputation strategies. Despite this, the number of studies testing imputation performance in aquaculture species is limited and they mainly use either FImpute or AlphaImpute software in Atlantic salmon (Kijas et al., 2017; Tsai et al., 2017; Yoshida et al., 2018; Kjetså et al., 2020; Tsairidou et al., 2020), rainbow trout (Vallejo et al., 2021; Yoshida et al., 2021) and Nile tilapia (Yoshida et al., 2019; Garcia et al., 2022). Only one recent study has tested Beagle imputation software in Atlantic salmon, common carp, sea bream and rainbow trout (Song and Hu, 2022). The promising results of these studies suggest that the combination of LD SNP panels with genotype imputation can achieve similar genomic prediction accuracies to HD panels. This combination can decrease the genotyping cost in aquaculture species, enabling the broader implementation of genomics in breeding programmes. However, in many cases the results of these studies are not directly comparable because they use different metrics to assess results and test different parameters. Therefore, further testing and optimisation of imputation algorithms and SNP selection methods is needed, across a range of aquaculture species and traits with the use of common assessment methods for genotype imputation to be routinely implemented in aquaculture selection programmes worldwide.

The objectives of this study were to i) evaluate the performance of three imputation software packages, FImpute v.3, AlphaImpute2 and findhap v.4 in breeding populations from four diverse aquaculture species; ii) investigate the impact of the number of markers in the LD panel and their selection method on imputation accuracy; and iii) evaluate the genomic prediction accuracy of imputed vs. LD genotypes for different traits in the four species. Our results contribute towards the definition of best practices for the broader application of genotype imputation and cost-effective genomic selection in aquaculture.

## 2 Materials and methods

### 2.1 Datasets

This study used previously published datasets from four species. Specifically:

• A farmed Atlantic salmon (*Salmo salar*) population of 624 individuals (90 parents and 534 offspring), belonging to 61 full-sib families as described in (Tsai et al., 2015). This population was challenged with *Lepeophtheirus salmonis* and sea lice counts on the fish were recorded for all the offspring. This trait had a positively skewed distribution and was logarithmically transformed. All individuals were genotyped with a 132 K SNP array, and 78,035 SNPs distributed across 29 pairs of chromosomes were retained after quality control for further analysis.
• A turbot (*Scophthalmus maximus*) population of 1,445 fish (47 parents and 1,398 offspring), distributed across 36 full-sib families as described in (Anacleto et al., 2019). The gonads of the fish were checked for the presence or absence of a parasite causing Scuticociliatosis (*Philasterides dicentrarchi*). Individuals were genotyped using RAD-seq and after quality control 11,069 SNPs were successfully mapped to the 22 pairs of chromosomes.
• A common carp (*Cyprinus carpio*) population of 1,319 individuals (60 parents and 1,259 offspring), comprising 195 full-sib families. This population was challenged with koi herpesvirus as described in Palaiokostas et al. (2018) and phenotypic records of body weight were obtained. Individuals were genotyped using RAD-Seq sequencing method and 15,615 SNPs were retained for downstream analysis (Palaiokostas et al., 2019). The positions of these markers were updated according to the latest reference genome (GenBank assembly accession number GCA_018340385.1) by using standard nucleotide BLAST (Altschul et al., 1990) and 8,506 SNPs were successfully assigned to 50 pairs of chromosomes from which 8,103 SNPs were retained after quality control.
• A Pacific oyster (*Crassostrea gigas*) population of 762 individuals (44 parents and 718 offspring), belonging to 30 full-sib families. Individuals in this study were challenged with ostreid herpesvirus (OsHV-1), measured for time to death, and genotyped using a SNP array with ∼27 K informative Pacific oyster SNPs (Gutierrez et al., 2020). After updating the SNP positions according to the latest genome assembly (Peñaloza et al., 2020) and quality control, 16,447 SNPs remained, distributed across the 10 chromosome pairs.

### 2.2 Quality control

All datasets were filtered using PLINK v.1.9 (Purcell et al., 2007). Individuals with just one of their two parents genotyped or >20% missing genotypes were excluded from the analysis. SNPs with >10% missing genotypes; significant deviation from Hardy–Weinberg Equilibrium (*p*-value < 10^−6^); MAF <0.05; or Mendelian error rates >10% were also excluded from subsequent imputation analyses. A summary of the data for the different species before and after quality control can be found in Table 1. After imputation, all the datasets were filtered again for MAF (<0.05).

**TABLE 1:** Summary of the datasets.

Species	SNPs before and after QC		Individuals before and after QC		Full-sib families	Phenotypes	Study with available dataset
*Salmo salar*	78,362	78,035	624	606	57	Sea lice (*Lepeophtheirus salmonis*) count	Tsai et al. (2015)
*Scophthalmus maximus*	17,690	11,069	1,445	1,396	38	Presence of parasites (*Philasterides dicentrarchi*) in the gonads	Anacleto et al. (2019)
*Cyprinus carpio*	8,506	8,103	1,319	1,172	195	Body weight	Palaiokostas et al. (2019)
*Crassostrea gigas*	22,994	16,447	762	701	30	Resistance to oyster herpesvirus (OsHV-1)	Gutierrez et al. (2020)

### 2.3 SNP selection methods for the low-density panels

The LD SNP panels were generated *in silico* by selecting 300, 500, 700, 1,000, 2,000, 3,000, 5,000 and 6,000 SNPs using the two methods described below. The LD panels were created by masking (i.e., setting to missing) all the SNPs not selected by each method.

#### 2.3.1 Physical-distance-based method

The selection of SNPs for the LD panels was implemented with a custom R script (available in https://github.com/Roslin-Aquaculture/Select-SNPs-to-generate-low-density-panels), considering the total number of SNPs and the length of each chromosome. For each density, a single panel was created with the number of markers selected being proportional to chromosome length and evenly distributed across the chromosomes according to position (physical distance). For this SNP selection method, the first and the last SNP on each chromosome were always selected and included in the LD panel. When no SNPs were available in the required position to achieve an even distribution, the closest available SNP was selected to obtain a LD panel with the desired number of markers. If a chromosome did not have enough SNPs (e.g., for densities ≥5,000 SNPs), all of the SNPs on that chromosome were selected and the final panel density was allowed to be slightly lower than expected (i.e., no additional SNPs were selected on the other chromosomes).

#### 2.3.2 Genetic-distance-based method

For the SNP selection method based on linkage disequilibrium, PLINK 1.9 (Purcell et al., 2007) was used to generate pruned SNP subsets based on variable window size, step size and squared correlation (r^2^) threshold values, to achieve the desired number of SNPs for each density. SNP pruning was performed using the “*--indep-pairwise*” command. In brief, at each step, squared correlation was calculated between each pair of SNPs within a genomic window, specified using SNP count (“variant ct”). All SNPs with squared correlation greater than the given r^2^ threshold were removed from the window until there were no such pairs. At the end of each step, the window was shifted forward by a “step size (variant ct),” and the procedure was repeated. A single LD panel was created for each target density.

#### 2.3.3 Randomly selected SNPs

Additionally, four LD panels were generated by randomly choosing 300, 500, 700 and 1,000 SNPs throughout the genome to test prediction accuracy before and after imputation with FImpute v.3.

### 2.4 Genotype imputation

Imputation of the offspring’s LD genotypes was performed using their parents as reference population (genotyped for the HD panels) with three software packages: AlphaImpute2 (Whalen and Hickey, 2020), FImpute v.3 (Sargolzaei et al., 2014) and findhap v.4 (VanRaden et al., 2013); a two-generation pedigree was available for all datasets, therefore pedigree and population-based imputation were performed.

AlphaImpute2 (Whalen and Hickey, 2020) imputation was performed separately for each chromosome using the default parameters, which are listed below, and SNPs in the genotype input file were ordered according to position on the chromosome. In the first step of pedigree imputation, five rounds of multi-locus iterative peeling were performed. The genotype calling threshold for the first round of peeling before phasing was 0.9. In the second step, where the algorithm builds the reference haplotype library, five rounds of phasing were conducted. Finally, for the third step of pedigree imputation another five rounds of multi-locus iterative peeling were performed, using the phased genotypes in the second step, and genotypes were set to the best-guess.

FImpute v.3 (Sargolzaei et al., 2014) uses a single genotype file with all the chromosomes present, and also requires information of the genomic location of the SNPs, provided in a map file, to model recombination. The “parentage_test” parameter was used to check for parentage errors with an error rate threshold of 0.05 to find progeny-parent mismatches. When a progeny-parent Mendelian inconsistency was detected, in most cases, genotypes of progeny and parents were set to missing and re-imputed. For this analysis, the conflicting parents were set to missing and original genotypes were not adjusted. In the results presented here, random filling of genotypes based on allele frequency was used to allow for a better comparison with AlphaImpute2.

For Findhap v.4 (VanRaden et al., 2013), the maximum and minimum length of haplotype segments were defined as 600 and 65, respectively, with an overlapping length of 10 and an error rate of 0.004. The number of different haplotypes within any segment was set to 1,000 for the lower densities, and it was increased to 2,000 for the 5,000 and 6,000 SNPs densities to consider all the possible haplotypes.

For all three methods, imputation accuracy was measured as the average Pearson correlation between the original and the imputed genotypes for each test individual. To test the effect of MAF on imputation accuracy, we calculated minor allele frequencies with PLINK v.1.9 and divided the SNPs into five MAF bins: (0–0.1], (0.1–0.2], (0.2–0.3], (0.3–0.4] and (0.4–0.5].

### 2.5 Estimation of genetic parameters

For each trait in the different datasets, heritabilities were estimated using ASReml 4.2 (Gilmour et al., 2021) using a linear mixed model as follows:

y=μ+Xb+Za+e

where y is a vector of observed phenotypes, μ is the overall mean of phenotype records, b is the vector of fixed effects, a is a vector of additive genetic effects distributed as $a∼N\left(0,G\sigma_{a}^{2}\right),$ where $\sigma_{a}^{2}$ is the additive genomic variance and G is the genomic relationship matrix, while X and Z are the corresponding incidence matrices for fixed and additive effects, respectively, and e is a vector of residuals.

Gonad parasite trait in the turbot dataset was binary, thus we used the generalized linear mixed model with the logit link function that links the probability of observing an event to the underlying linear model:

$$P\left(y_{i}=1\right)=\frac{\exp\left(\mu +{Xb}_{i}+{Za}_{i}+e_{i}\right)}{1+\exp\left(\mu +{Xb}_{i}+{Za}_{i}+e_{i}\right)}$$

The fixed effects included in the different models for each species were i) body weight in Atlantic salmon, ii) factorial-cross group (four levels) in carp, iii) box (36 levels) in turbot, and iv) tank (two levels) in oyster.

The genomic relationship matrix between pairs of individuals j and k (*g*_*jk*_) was calculated using the GCTA software (Yang et al., 2011) as follows:

$$g_{jk}=\frac{1}{N}\sum_{i=1}^{N}\frac{\left(x_{ij}-{2p}_{i}\right)\left(x_{ik}-{2p}_{i}\right)}{{2p}_{i}\left(1-p_{i}\right)}$$

where N is the total number of SNPs, $x_{ij}$ and $x_{ik}$ are the number of copies of the reference allele for the $i^{th}$ SNP for the $j^{th}$ and $k^{th}$ fish, respectively, and $p_{i}$ is the frequency of the reference allele estimated from the markers.

### 2.6 Cross-validation for genomic -based prediction accuracy

The accuracy of genomic prediction was estimated by 20 replicates of fivefold cross-validation analysis (80% of individuals in the training set and 20% in the validation set; “CVrep” GitHub statistical R package (Tsairidou 2019), available at https://github.com/SmaragdaT/CVrep). The phenotypes in the validation set were masked, and genomic best linear unbiased prediction (GBLUP) was applied to predict the breeding values of the validation set individuals in ASReml 4.2 (Gilmour et al., 2021), using the linear mixed model described above. Prediction accuracy was calculated as the correlation between the predicted breeding values of the validation set and the actual phenotypes divided by the square root of heritability, estimated from the full dataset for each trait [ $\approx \frac{r\left(y,ŷ\right)}{h}$ ].

## 3 Results

### 3.1 Trait summary and genetic parameters

A different phenotype was used in each dataset (Table 2): i) In Atlantic salmon, log-transformed sea lice counts were used as phenotype. Log-transformed sea lice counts had a mean of 3.11 ± 0.56 and a genomic heritability estimate of 0.19 ± 0.07. ii) In turbot, the binary trait of absence or presence of gonad parasites was used. Gonad parasites were present in 881 individuals, while 441 individuals were free of parasites. The estimated genomic heritability for this trait was 0.27 ± 0.08. iii) In Pacific oyster, we used the phenotype of days to death after infection with OsHV-1-μvar, with survivors being assigned a value of 8 days (end of the challenge). The mean and standard deviation of surviving days was 6.91 ± 1.82, and the estimated genomic heritability was 0.64 ± 0.05. iv) In common carp, the mean value for body weight was 16.36 ± 4.65 g, and the heritability estimate was 0.22 ± 0.04.

**TABLE 2:** Genomic heritability and prediction accuracy using HD panels.

Species	Phenotypes	Genomic heritability estimates	HD panel genomic prediction accuracy (mean ± sd)
Atlantic salmon	Log transformed sea lice (*Lepeophtheirus salmonis*) count	0.19 ± 0.07	0.54 ± 0.05
Turbot	Presence/absence of gonad parasites (*Philasterides dicentrarchi*)	0.27 ± 0.08	0.34 ± 0.02
Common carp	Body weight	0.22 ± 0.04	0.69 ± 0.02
Pacific oyster	Resistance to oyster herpesvirus (OsHV-1) measured as time to death	0.64 ± 0.05	0.62 ± 0.03

### 3.2 Accuracy of imputation

**FIGURE 1:**
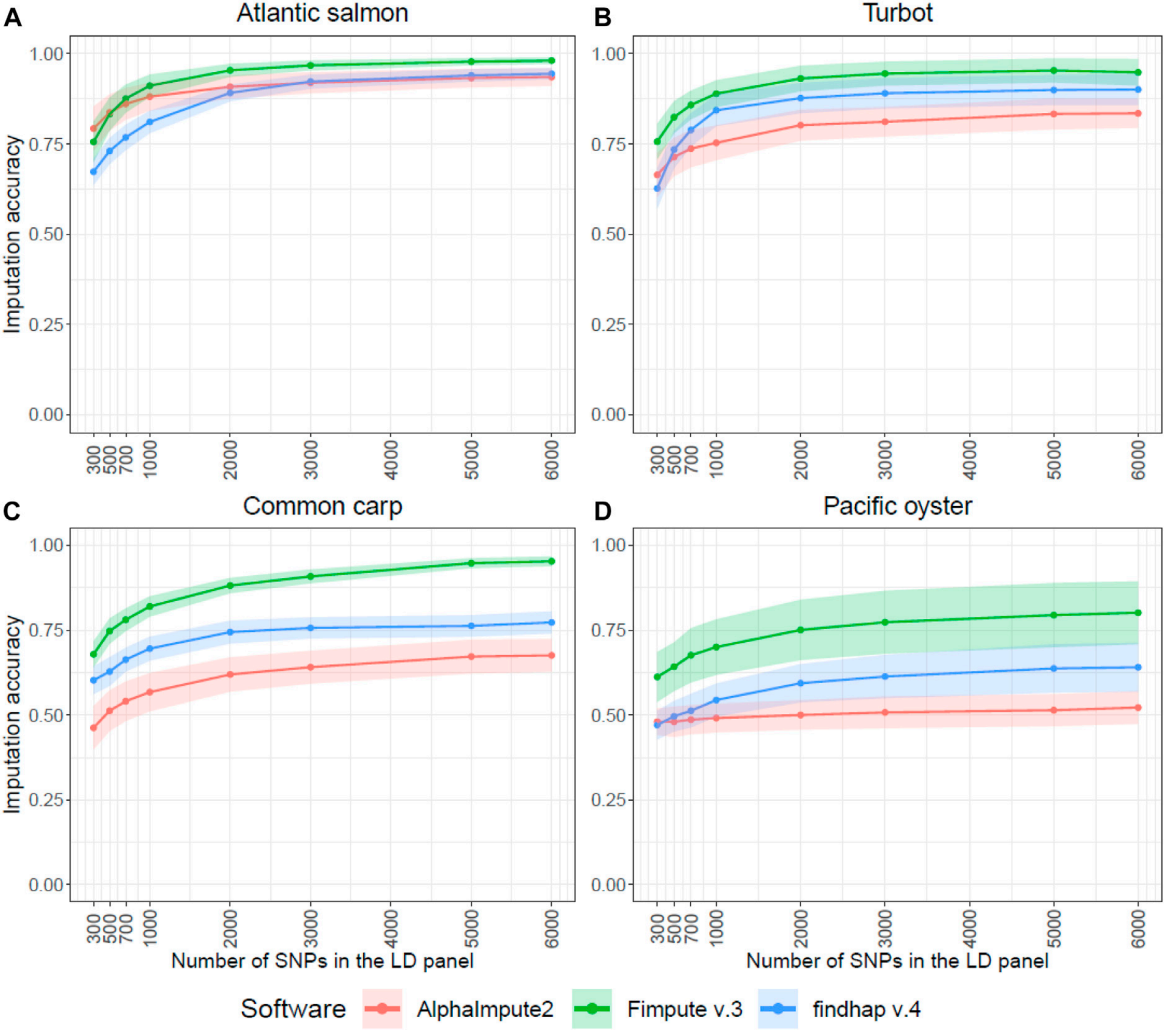
Genotype imputation accuracy in four aquaculture species. Average genotype imputation accuracy (correlation between true and imputed genotypes) for the three imputation software in each of the four species. The ribbons represent the standard deviation of the average imputation accuracy across all individuals. The SNP selection method based on physical distance was used to impute the LD panels in these graphs. The Atlantic salmon LD panels **(A)** were imputed to 78,035 SNPs, the turbot **(B)** to 11,069 SNPs, the common carp **(C)** to 8,103 SNPs and the Pacific oyster **(D)** to 16,447 SNPs.

Imputation accuracy increased with increasing panel density for all software (Figure 1). Overall, the results revealed that FImpute v.3 was more accurate for most of the densities in all the species, and findhap v.4 was mostly second in the ranking. Although AlphaImpute2 was generally ranked last between the three software, it outperformed findhap v.4 in terms of accuracy for the five lowest densities (300–2,000 SNPs) in the Atlantic salmon dataset. It also outperformed FImpute v.3 at the lowest density of 300 SNPs (Figure 1A). Imputation accuracy for the lowest density of 300 SNPs, when imputing with FImpute v.3, ranged between 0.61 (Pacific oyster) and 0.76 (Atlantic salmon and turbot). For the 6,000 SNPs density, the fish species reached very high imputation accuracies (0.95–0.98), but the accuracy value was noticeably lower for Pacific oyster (0.80) (Figure 1).

Regarding computing time, FImpute v.3 was faster than the other two software tested. Running time results of the three software when imputing the 300 SNPs panel density for each species are shown in Table 3. The average computational time across the four species for the LD panel of 300 SNPs when imputing with FImpute v.3 was 1 min and 13 s, with findhap v.4 showing a similar average running time of 1 min 55 s, and AlphaImpute2 considerably longer running times of 24 min 56 s in average.

**TABLE 3:** Computational time for each software to impute from the 300 SNPs density panel.

Species	FImpute v.3	Findhap v.4	AlphaImpute2
*Salmo salar*	1 min 16 s	2 min 49 s	30 min 36 s
*Scophthalmus maximus*	47 s	1 min 45 s	16 min 46 s
*Cyprinus carpio*	27 s	1 min 17 s	44 min 01 s
*Crassostrea gigas*	1 min	1 min 7 s	7 min 41 s

**FIGURE 2:**
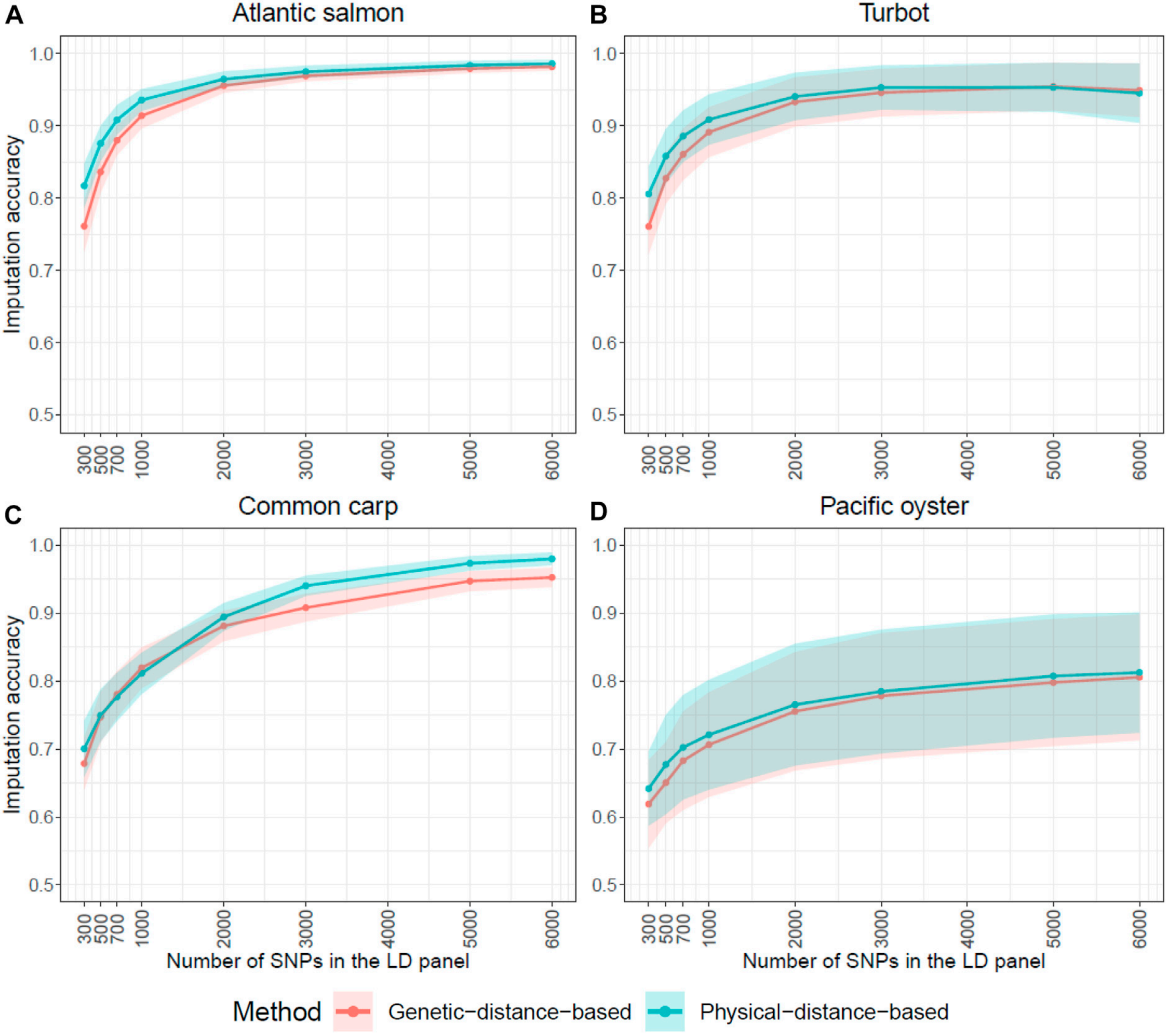
Influence of LD SNP panel design on imputation accuracy. Average genotype imputation accuracy (correlation between true and imputed genotypes) using FImpute v.3 in each of the four species for the two SNP selection methods: physical and genetic distance-based. The ribbons represent the standard deviation of the average imputation accuracy across all individuals. The y-axis in these graphs ranges from 0.5 to 1 to facilitate the comparison of the two methods.

In Figure 2, the genetic distance method based on linkage disequilibrium slightly increased the accuracy of imputation for most of the very low densities in Atlantic salmon, turbot and Pacific oyster (300–2,000 SNPs), while in the common carp dataset it improved the imputation accuracy of the higher densities (2,000–6,000 SNPs) (Figure 2). However, the differences observed in imputation accuracy between the two LD panel SNP selection methods were mostly non-significant. Since both the imputation and prediction accuracy results of the imputed panels were similar when the SNPs were selected with the genetic or the physical-distance-based method, the results we present below are with the physical-distance-based-method and imputed with FImpute v.3 software package.

**FIGURE 3:**
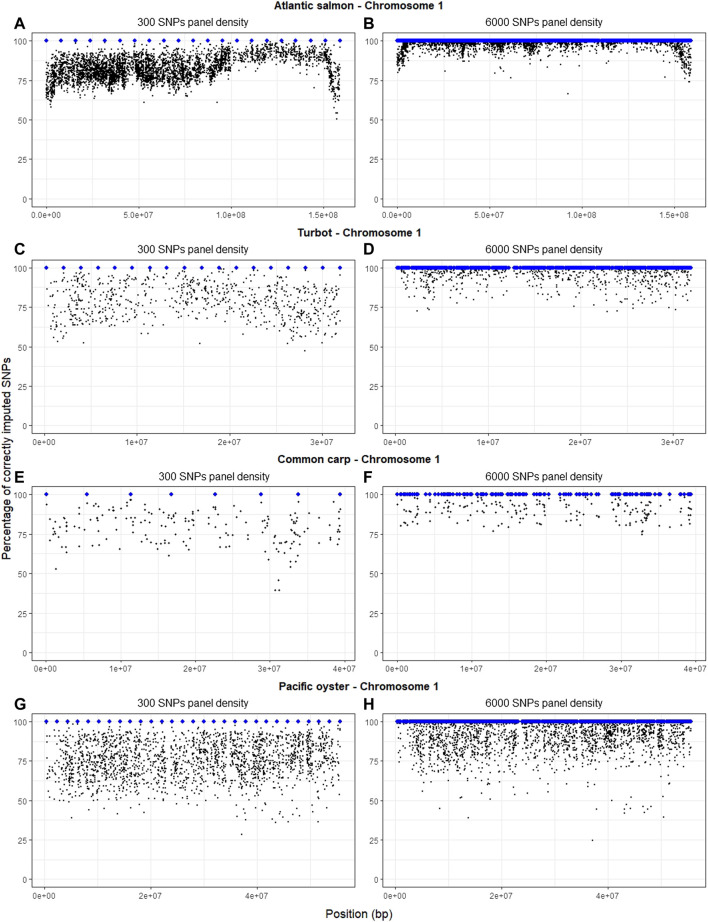
Percentage of correctly imputed genotypes with FImpute v.3 for each SNP of chromosome 1 in each of the four species, using the LD panels of 300 **(A,C,E,G)** and 6,000 **(B,D,F,H)** SNPs (selected with the physical-distance-based method). The blue dots indicate the physical position of the SNPs in the LD panel, whereas the black dots indicate the imputed SNPs.

There is a visible pattern of slightly decreased imputation accuracy at the ends of the chromosomes of the four species (Figure 3), but this was not consistent for all chromosomes (Supplementary Material). This phenomenon is clearer in Atlantic salmon (Figures 3A, B), possibly due to the higher number of SNPs in the HD panel. Increasing the SNP density of the LD panel from 300 SNPs to 6,000 SNPs substantially improved imputation accuracy throughout the chromosome and especially at chromosomal ends (Figure 3). In the oyster dataset, there were poorly imputed SNPs throughout the chromosome, and for some of these SNPs accuracy did not improve when the panel density was increased (Figures 3G, H).

**FIGURE 4:**
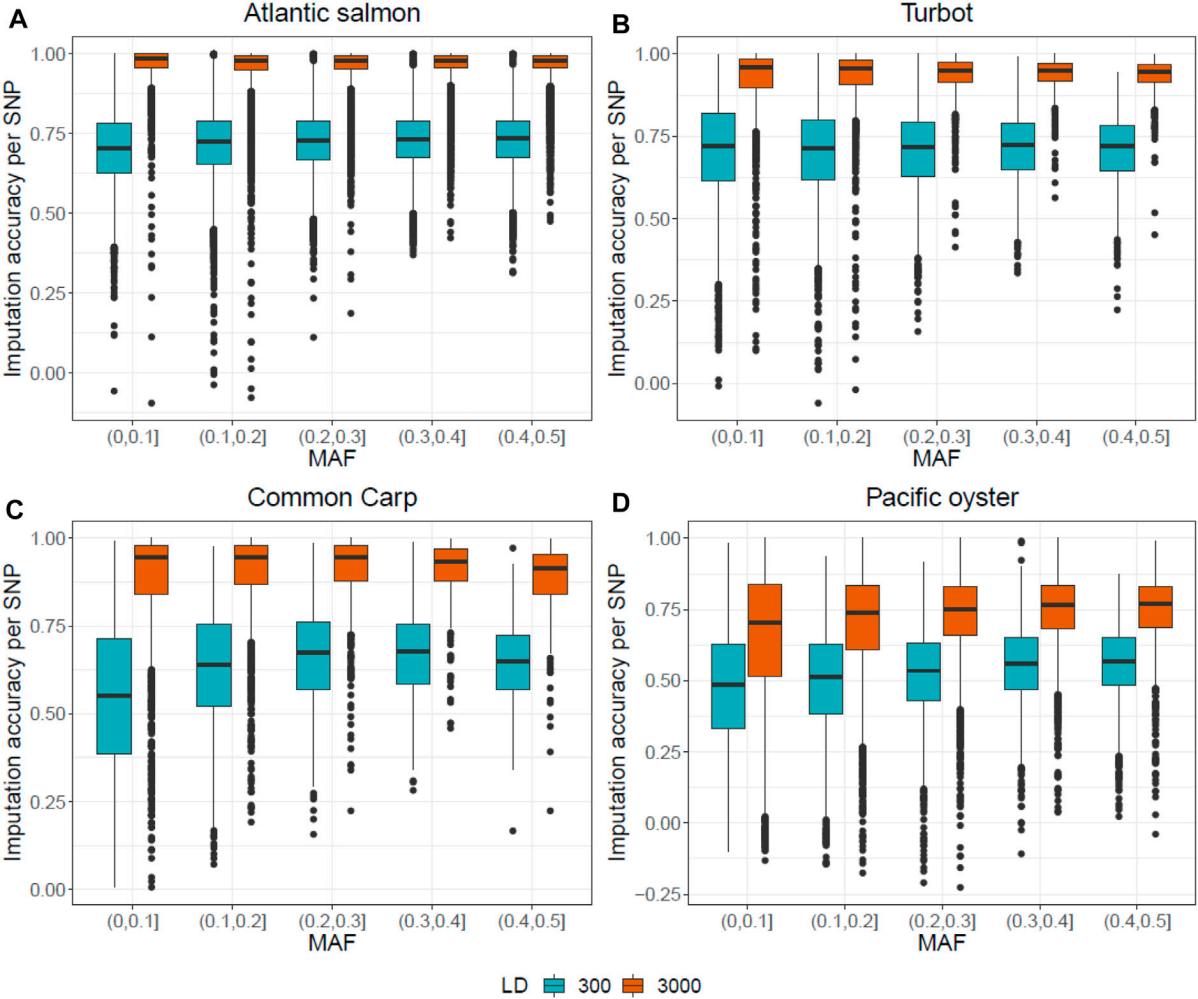
Correlation between the original and the imputed genotypes for each SNP plotted against MAF, for the two LD panels of 300 and 3,000 SNPs. Genotypes of the Atlantic salmon **(A)**, turbot **(B)**, common carp **(C)** and Pacific oyster **(D)** dataset were imputed with FImpute v.3.

Figure 4 shows the effect of MAF on imputation accuracy using FImpute v.3. The density of the LD panel did not seem to have a MAF-dependant impact on the imputation accuracy. However, there is a wider distribution of imputation accuracy values in the (0–0.1) MAF bin compared to the other bins, suggesting that there were more SNPs with very low MAF that were poorly imputed.

### 3.3 Genomic prediction using imputed SNP panels

**FIGURE 5:**
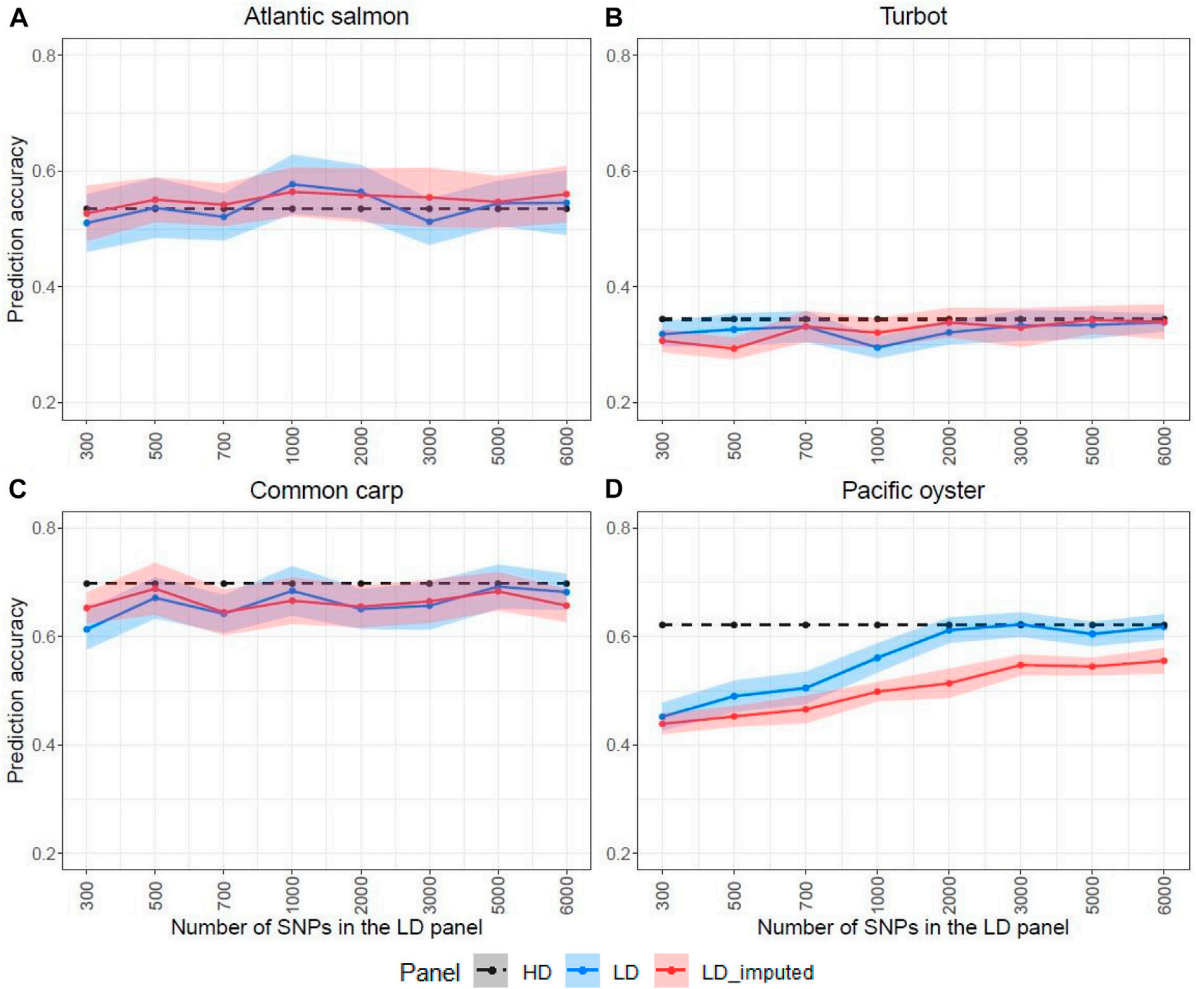
Prediction accuracies estimated for the high-density (HD), the low-density (LD) and the imputed LD panels (LD-imputed) for the four species. The LD panels were designed with the physical-distance-based method. The ribbons represent the standard deviations over 20 replicates of fivefold cross-validation analyses. The y-axis in these graphs ranges from 0.2 to 0.8 to facilitate the comparison between the LD and LD-imputed prediction accuracies. The Atlantic salmon LD panels **(A)** were imputed to 78,035 SNPs, the turbot **(B)** to 11,069 SNPs, the common carp **(C)** to 8,103 SNPs and the Pacific oyster **(D)** to 16,447 SNPs with FImpute v.3 software.

The HD panel was used to estimate the genomic heritability and obtain genomic prediction accuracies for each species (Table 2), which were compared to those obtained using the LD panels (Figure 5). Prediction accuracies were estimated for the LD panels with and without imputation. For Atlantic salmon, turbot and common carp, genomic prediction using the LD and the imputed panels gave comparable accuracies, which were very close to the accuracies obtained with the HD panel (Figures 5A–C). However, in the Pacific oyster, all the LD panels (300–6,000 SNPs) outperformed the imputed panels (Figure 5D), reaching maximal prediction accuracy when the LD panel consisted of 2,000 SNPs.

**FIGURE 6:**
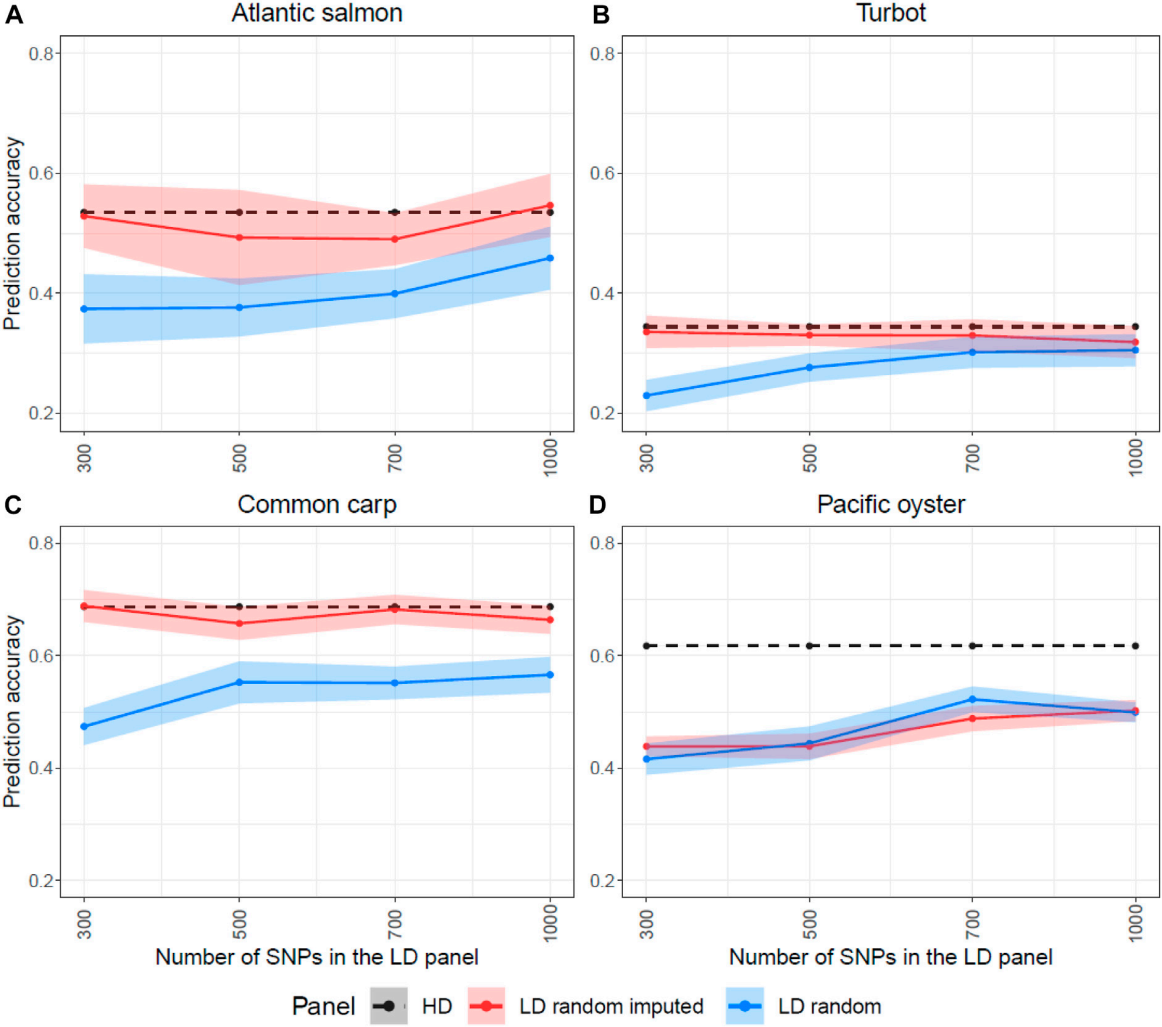
Prediction accuracies estimated for the high-density (HD), the low-density (LD random) and the imputed LD panels (LD random imputed), when SNPs were randomly selected for the four species. The y-axis in these graphs ranges from 0.2 to 0.8 to facilitate the comparison. The Atlantic salmon LD panels **(A)** were imputed to 78,035 SNPs, the turbot **(B)** to 11,069 SNPs, the common carp **(C)** to 8,103 SNPs and the Pacific oyster **(D)** to 16,447 SNPs with FImpute v.3 software.

Since these results were unexpected according to previous reports, which showed that the accuracy of genomic prediction post imputation was higher than using the LD panels, we wanted to further investigate whether the SNP selection methods were responsible for the high prediction accuracy of the LD panels without imputation. Therefore, we randomly sampled SNPs throughout the genome to generate LD panels and perform imputation to compare their prediction accuracy with the other SNP selection methods. Figure 6 shows the prediction accuracy of four LD panels (300, 500, 700 and 1,000 SNPs) with and without imputation. For all four species, the prediction accuracy of the randomly designed LD SNP panels was considerably lower than the accuracy achieved with the HD SNP panel. Imputation of these LD SNP panels improved the predictive ability for Atlantic salmon, turbot and common carp with accuracy values very close to the maximal. However, the imputation of the Pacific oyster’s random LD SNP panel did not improve prediction accuracy. Both the randomly designed LD panel and the imputed one achieved similar results that were lower than the accuracy of the HD panel (Figure 6D).

## 4 Discussion

Genotype imputation is a powerful tool that has the potential to reduce the genotyping cost of genomic selection in aquaculture breeding programmes without a dramatic loss of prediction accuracy. In this study, we investigated some of the main factors affecting the accuracy of imputation and genomic prediction to contribute towards the establishment of best practices for the wider application of this method in the aquaculture sector.

### 4.1 Choice of imputation software

Three genotype imputation software were tested for their performance and compared between four populations of different aquatic species. All three software packages used a combination of population and pedigree-based imputation methods, and both parents’ genotypes were present for all individuals in the datasets. The existence of pedigree information and close relatives in the dataset becomes more important as the number of markers in the LD panels decreases, as it becomes difficult to find the truly shared haplotypes between the reference and the target individuals.

FImpute showed the best performance across the four species in our study, with highest imputation accuracies for most LD panels and a shorter running time. FImpute shows extremely fast computational times when compared to other imputation software (e.g., Beagle, findhap, AlphaImpute, PHASEBOOK, Eagle-Minimac4 approach) for populations where pedigree information was available (Johnston et al., 2011; Chud et al., 2015; Ventura et al., 2016; Wang et al., 2016; Pausch et al., 2017; Ye et al., 2018; Fernandes Júnior et al., 2021). Compared to AlphaImpute2, which uses a probabilistic algorithm (Whalen and Hickey, 2020), FImpute and findhap are faster in speed because they directly search for haplotypes in descending size and frequency order (Vanraden et al., 2011). FImpute is also known to infer rare alleles with higher accuracy (Ma et al., 2013; Wang et al., 2016; Fernandes Júnior et al., 2021) because the process starts by effectively matching long haplotypes between closely related individuals (Sargolzaei et al., 2014). This is pertinent because in a population with closely related individuals, the long haplotypes shared between them usually carry rare alleles (Kamatani et al., 2004) which can be frequent in families with a common ancestor who had the variant (Liu et al., 2019).

### 4.2 Composition of the low-density panels

The number of SNPs in the LD panel and the linkage disequilibrium between adjacent SNPs was found to substantially affect imputation accuracy; by increasing the number of SNPs in the LD panels, we observed an increase in imputation accuracy (Figure 1). As previously discussed by Sargolzaei et al. (2014) this is because it becomes more likely to find shorter haplotype segments shared between related individuals due to the improved crossover resolution (Sargolzaei et al., 2014). However, there was a number of SNPs in the LD panel above which imputation accuracy improved only slightly (Figure 1). For Atlantic salmon, turbot and Pacific oyster the number of SNPs to reach this plateau was between 2,000 and 3,000.

In Pacific oyster, imputation accuracy was lower for all the LD panels compared to the fish species. Previous studies have found that some Pacific oyster populations exhibit rapid decay of linkage disequilibrium (Gutierrez et al., 2017; Zhong et al., 2017). This means that recombination between markers at each generation is high and therefore higher SNP densities might be required to achieve the same imputation accuracy results achieved in the other species. Additionally, the oyster genome, and in general bivalves’ genomes, is highly polymorphic. Studies have shown that the Pacific oyster genome exhibits high levels of heterozygosity and is abundant in repetitive sequences, with some active transposable elements shaping this genomic variation (Zhang et al., 2012; Hedgecock et al., 2015; Gutierrez et al., 2017). These highly polymorphic regions hinder the construction of the genome assembly (Gutierrez et al., 2020) and can lead to a pronounced decrease in imputation accuracy (Fernandes Júnior et al., 2021), possibly due to errors in marker order. Other characteristics of their genome that may be impairing mapping and consequently imputation accuracy are the putative high rate of *de novo* mutations during meiosis or larval development, which contribute to unusual segregation patterns and deviations from Mendelian inheritance patterns (Hedgecock et al., 2015; Soledad Peñaloza Navarro, 2017). Imputation of bivalve genomes requires further research in different populations and species to discover which parameters can contribute towards the improvement of imputation accuracy and their resulting prediction accuracy.

Regarding chromosomal position, we observed a lower number of correctly imputed SNPs at the beginning and at the end of Atlantic salmon chromosome 1. However, this decreased imputation accuracy at chromosomal ends was not evident in all the species. The lower number of SNPs available in the HD panel for some species may have had an effect in our ability to discern drops in imputation accuracy in certain regions of the genome; recombination and linkage disequilibrium can also explain the differences in imputation accuracy. Poorly imputed SNPs can be found in chromosomal regions with high recombination rates (Hozé et al., 2013), such as the beginning and the end of chromosomes in some species (Druet et al., 2010; Ventura et al., 2016), or in regions difficult to assemble, but it can also be related to patterns of linkage disequilibrium throughout the genome. For example, recombination hot spots make the precise reconstruction of haplotypes difficult; consequently, imputation accuracy is low in these regions (Yoshida et al., 2018). Centromeres also tend to show low imputation accuracies because they are difficult to assemble, potentially leading to incorrect order of markers. If we exclude centromeres and telomeres, regions with high imputation errors can be related to the patterns of linkage disequilibrium throughout the genome. SNPs with incorrect positions on the genetic map or SNPs wrongly assigned to chromosomes are challenging to impute, because they are not in linkage disequilibrium with the neighbouring markers on the map (Druet et al., 2010; Yoshida et al., 2018). Overall, as the density of the LD panels increased, imputation accuracy at the extremes and throughout the chromosomes increased due to the increased resolution of recombination patterns (Yoshida et al., 2018; Fernandes Júnior et al., 2021).

### 4.3 Genomic prediction accuracy

Low-cost genomic selection is successful when the genotype data of LD panels accurately capture the genetic variation among the training and prediction individuals, resulting in no or minor loss of prediction accuracy when compared to HD genotypes. In this study, we achieved highly accurate genomic breeding value estimates for SNP densities as low as 300 SNPs for the Atlantic salmon, turbot and common carp populations. Small numbers of markers were sufficient probably because the shared haplotypes and linkage blocks between the reference and target individuals are long (full and half-sibs of the test population present in the reference population), and therefore their effects can be captured even with a small number of markers. Further, the number of families in a standard aquaculture breeding programme is small (100–200 families). The small effective population size and the degree of relatedness between individuals can explain the good performance of extremely low-density SNP panels.

Other studies have shown that a small number of markers and imputation are sufficient for accurate genomic prediction. For example, Gorjanc et al. (2017) suggested that 200 SNPs (20 SNPs per chromosome for a 10 chromosome simulated genome of 20,000 SNPs in total) imputed to HD can result in prediction accuracies comparable to HD panels in plant populations with a structure similar to that of aquaculture populations. Delomas et al. (2023), in a simulation study in oysters, achieved nearly maximal accuracy of genomic estimated breeding values by using 250–500 LD panels imputed to 40,000 SNPs. However, we did not observe similar high prediction accuracy results in our study with the Pacific oyster population we tested. In a study in Atlantic salmon, imputed genotype data from a ∼250 LD SNP panel achieved comparable genomic prediction accuracy results to the true genotype data in Tsai et al. (2017); Yoshida et al. (2018) studied a two-generation Atlantic salmon population and suggested a genotyping strategy which combines genotyping all the parents and 10% of offspring with a HD panel, while the rest of the progeny are genotyped with a 500 SNPs panel and imputed to HD to achieve identical genomic prediction accuracies as with the 50,000 SNP panel. In another Atlantic salmon study, genotyping offspring at the very LD of 200 SNPs and imputing them with FImpute 2.2 to their parents’ medium-density panel (5,000 SNPs) achieved almost the same genomic prediction accuracy as the true medium-density panel (Tsairidou et al., 2020). There is a general consensus that imputation leads to close to maximal prediction accuracy.

Our findings demonstrate that for three out of the four species tested, the accuracy of genomic prediction is heavily dependent on the choice of SNPs when using the LD panels without imputation. The selection of evenly distributed SNPs in the LD panels resulted in markedly higher prediction accuracies when compared to that obtained with randomly selected SNPs. Whilst evenly distributed SNPs did not benefit from imputation, since the accuracy was already similar to that obtained with HD panels, imputation significantly increased the accuracy of randomly selected LD panels, bringing it in line with HD genotypes. In conclusion, the choice of SNPs in the LD panel is crucial when they are used without imputation for genomic selection; however, if imputation is used the choice of SNPs in the panel is irrelevant. Considering that the LD panel would have to be designed specifically for the target population, and that its performance might decrease as the genetic makeup of the population changes with each generation of selection, imputation is an exceptional tool to ensure that near-maximum prediction accuracies are obtained in every scenario.

Imputation accuracy did not affect prediction accuracy in the three fish species tested, with imputation accuracies of 0.76–0.98 depending on the number of SNPs in the LD panel resulting in similar prediction accuracies. However, this is not true in oysters, where the prediction accuracy of the imputed LD panel was significantly lower than that achieved with the LD panel alone, even when the number of SNPs in the LD panel was increased to 6,000 (Figure 5D). In this dataset, imputation accuracy was lower compared to the other species (Figure 1), which can probably explain why the LD panels outperformed the imputed panels. Because of the rapid decay of linkage disequilibrium in the Pacific oyster, breeding candidates require regular testing on close relatives to preserve high accuracy levels between generations in a breeding programme (Gutierrez et al., 2020). Nonetheless, more studies in bivalve species are necessary to determine if this is a general phenomenon or rather specific to the dataset studied here.

### 4.4 Cost reduction by using LD panels and genotype imputation

A significant cost reduction can be achieved by sequencing the target population with a very low-density panel (300–500 SNPs), which should still provide maximal prediction accuracy when combined with imputation to HD, using a reference population containing relatives of the target population. While using the LD panels alone could result in a further reduction of the cost of genomic selection, we consider that the potential risk is not worth it since the number of animals that have to be genotyped at HD for imputation is low (i.e., the number of animals in aquaculture broodstock populations is usually around 100). In any case, if we estimate the cost of HD genotyping at $15 and the cost of LD genotyping at $12, for a relatively small population of 5,000 animals, the use of LD panels would result in a reduction of the cost in the application of genomic selection of 20% ($75,000 vs. $60,000). Considering that the cost of HD genotyping is usually higher for most aquaculture species and that most species require the use of genetic tools to reconstruct the pedigree, LD panels and imputation can play an important role in the incorporation of genomic selection into aquaculture breeding programmes worldwide.

## 5 Conclusion

In this study, we explored the use of LD panels and imputation to reduce the cost of genomic selection in aquaculture breeding programmes, exploring different imputation software and SNP selection methods. Imputation accuracies were very high for the three fish species tested, while the performance of imputation was markedly lower in our oyster dataset. FImpute v.3 was the fastest and most accurate imputation method in almost all scenarios tested. When the LD panels were used without imputation, LD panels with the SNPs evenly distributed across the chromosomes achieved prediction accuracies very similar to the HD panel in the three fish species, even with just 300 SNPs, while randomly selected LD panels resulted in markedly lower prediction accuracies. However, imputation significantly increased the prediction accuracy of the randomly selected LD panels, reaching values similar to those of the HD panel in the fish species. Our results indicate that genotyping cost for the implementation of genomic selection can be reduced by the use of LD panels or a combination of LD panels and imputation. While the use of appropriately selected LD SNP panels would be more cost-effective, we suggest the use of imputation to eliminate the risk from potential changes in performance of the LD panels. This manuscript will help facilitate the widespread adoption of genomic selection in commercial aquaculture, leading to increased production and stability.

## Data Availability

The datasets presented in this study can be found in online repositories. The names of the repository/repositories and accession number(s) can be found in the article/Supplementary Material.
