# Supplementary material for: Evaluation of low-density SNP panels and imputation for cost-effective genomic selection in four aquaculture species
Source: Front Genet. 2023 May 11;14:1194266. doi: 10.3389/fgene.2023.1194266 (PMC10213886; doi:10.3389/fgene.2023.1194266)
Supplement: Supplementary file 1 [file Image1.pdf]

Supplementary Material

Evaluation of low-density SNP panels and imputation for cost-effective genomic selection in four aquaculture species

Kriaridou, C.[1], Tsairidou, S.[2], Fraslin, C.[1], Gorjanc, G.[1], Looseley, M.[3], Johnston, I. A.[3], Houston, R.D.[1, 4] and Robledo, D.[1],*

1. The Roslin Institute, University of Edinburgh, UK

2. Global Academy of Agriculture and Food Systems, University of Edinburgh, UK

3. Xelect Ltd, UK

4. Benchmark Genetics, 1 Pioneer Building, Edinburgh Technopole, Penicuik, EH26 0GB, United Kingdom

*** Correspondence:** Corresponding Author: diego.robledo@roslin.ed.ac.uk

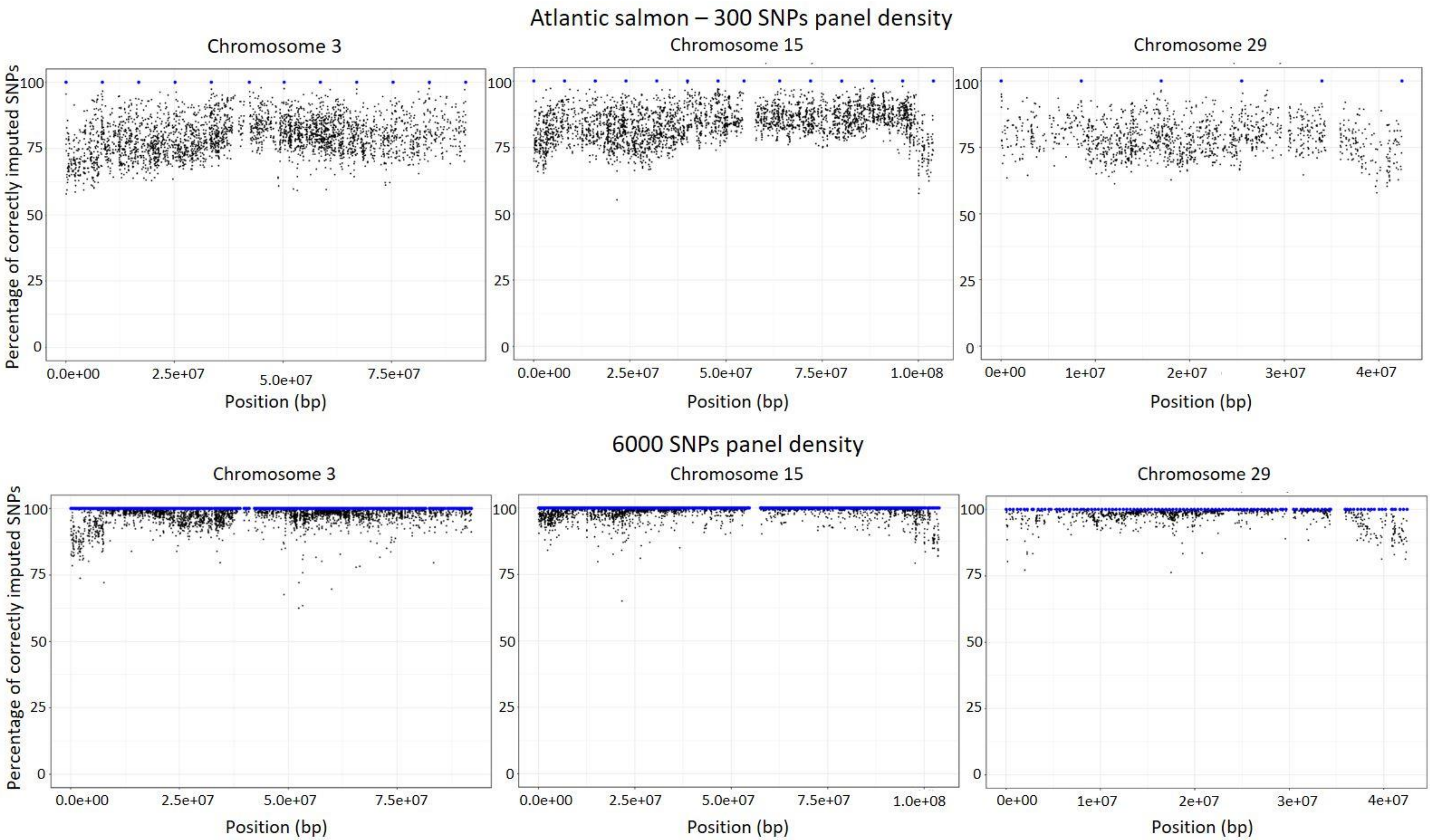

Supplementary figure 1. Percentage of correctly imputed genotypes with FImpute v.3 for each SNP of chromosome 3, 15 and 29 in Atlantic salmon dataset, using the LD panels of 300 and 6,000 SNPs (selected with the genetic-distance-based method). The blue dots indicate the physical position of the SNPs in the LD panel, whereas the black dots indicate the imputed SNPs.

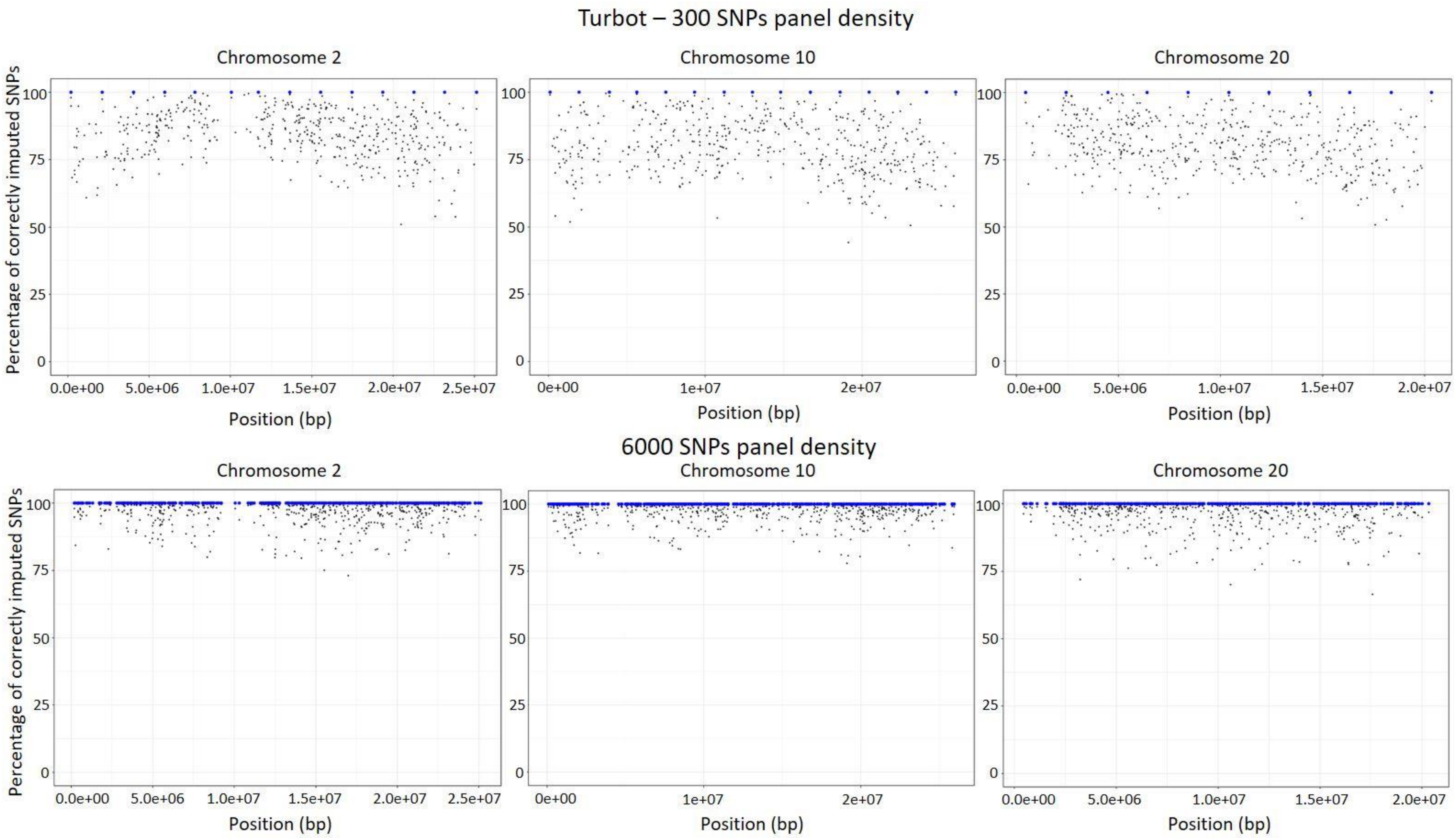

Supplementary figure 2. Percentage of correctly imputed genotypes with FImpute v.3 for each SNP of chromosome 2, 10 and 20 in turbot dataset, using the LD panels of 300 and 6,000 SNPs (selected with the genetic-distance-based method). The blue dots indicate the physical position of the SNPs in the LD panel, whereas the black dots indicate the imputed SNPs.

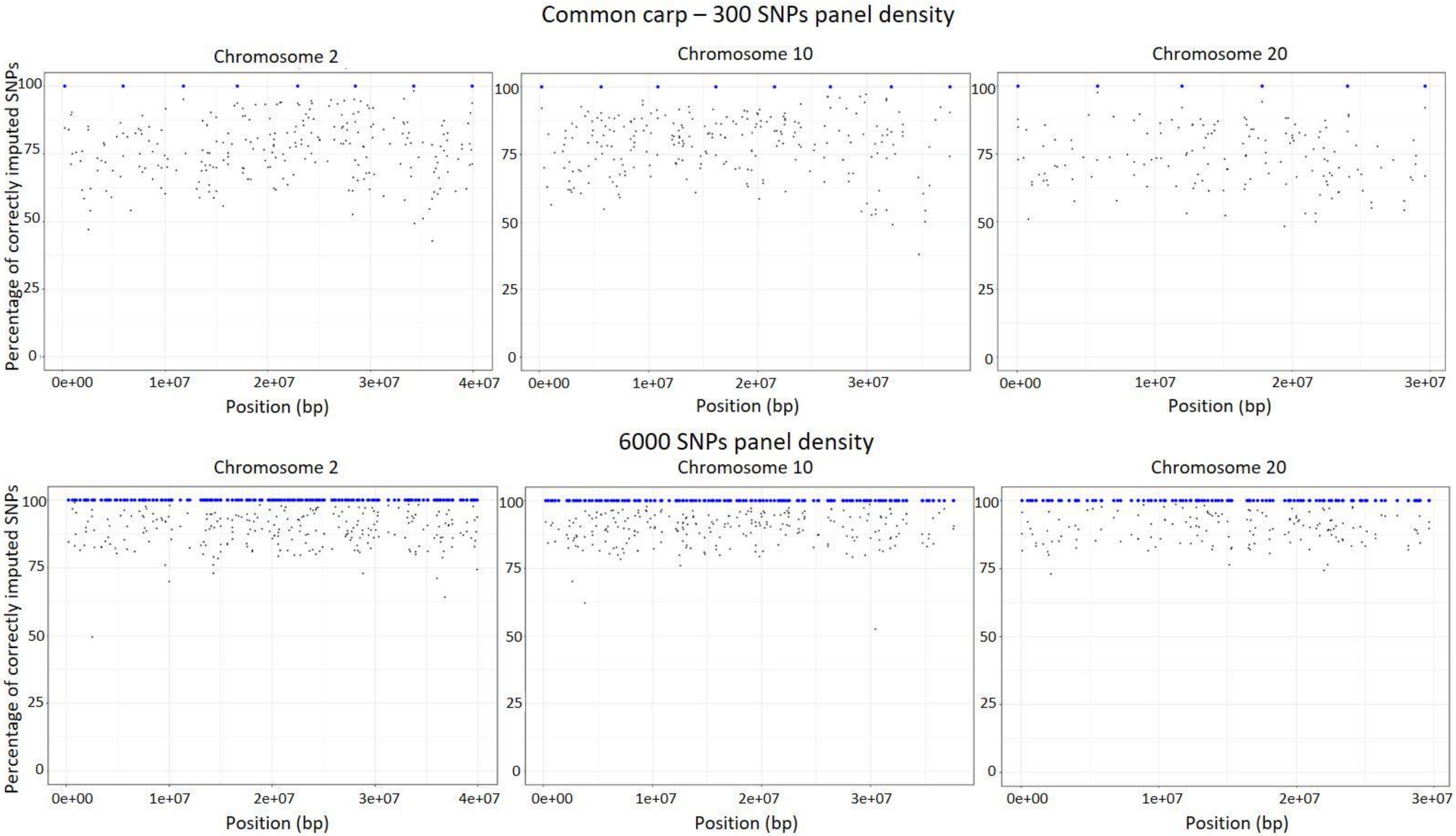

Supplementary figure 3. Percentage of correctly imputed genotypes with FImpute v.3 for each SNP of chromosome 2, 10 and 20 in the common carp dataset, using the LD panels of 300 and 6,000 SNPs (selected with the genetic-distance-based method). The blue dots indicate the physical position of the SNPs in the LD panel, whereas the black dots indicate the imputed SNPs.

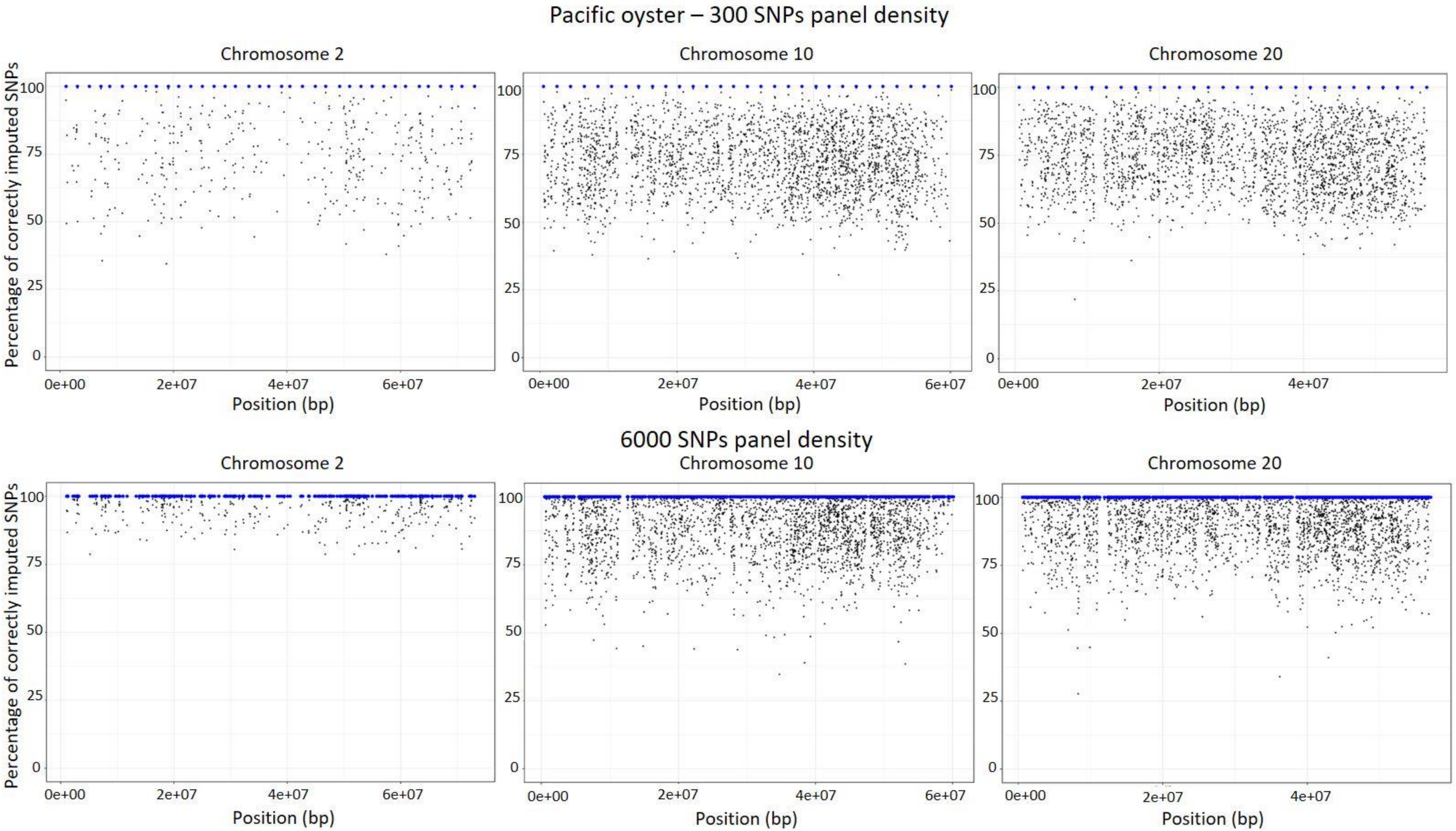

Supplementary figure 4. Percentage of correctly imputed genotypes with FImpute v.3 for each SNP of chromosome 2, 10 and 20 in the Pacific oyster dataset, using the LD panels of 300 and 6,000 SNPs (selected with the genetic-distance-based method). The blue dots indicate the physical position of the SNPs in the LD panel, whereas the black dots indicate the imputed SNPs.

Supplementary table 1. The datasets presented in this study were previously published and their availability status can be found in the articles mentioned below.

Species	Study with available dataset	DOI
Salmo salar	Tsai *et al*. 2015	https://doi.org/10.1186/s12864-015-2117-9
Scophthalmus maximus	Anacleto *et al*. 2019	https://doi.org/10.1038/s41598-019-40567-w
Cyprinus carpio	Palaiokostas *et al*. 2019	https://doi.org/10.3389/fgene.2019.00543
Crassostrea gigas	Gutierrez *et al*. 2020	https://doi.org/10.1111/age.12909
